# Nicotianamine Synthesis by *OsNAS3* Is Important for Mitigating Iron Excess Stress in Rice

**DOI:** 10.3389/fpls.2019.00660

**Published:** 2019-06-04

**Authors:** May Sann Aung, Hiroshi Masuda, Tomoko Nozoye, Takanori Kobayashi, Jong-Seong Jeon, Gynheung An, Naoko K. Nishizawa

**Affiliations:** ^1^Research Institute for Bioresources and Biotechnology, Ishikawa Prefectural University, Ishikawa, Japan; ^2^Department of Biological Production, Faculty of Bioresource Sciences, Akita Prefectural University, Akita, Japan; ^3^Center for Liberal Arts, Meiji Gakuin University, Kanagawa, Japan; ^4^Department of Global Agricultural Sciences, The University of Tokyo, Tokyo, Japan; ^5^Crop Biotech Institute and Graduate School of Biotechnology, Kyung Hee University, Yongin, South Korea

**Keywords:** iron excess, rice, OsNAS3, nicotianamine, deoxymugineic acid, detoxification

## Abstract

Iron (Fe) toxicity in plants causes tissue damage and cellular homeostasis disorders, thereby affecting plant growth and development. Nicotianamine (NA) is a ubiquitous chelator of metal cations and is responsible for metal homeostasis. Rice has three NA synthase (*NAS*) genes, of which the expression of *OsNAS1* and *OsNAS2* but not of *OsNAS3* is strongly induced in response to Fe deficiency. Recently, we found that *OsNAS3* expression is strongly induced with excess Fe in most rice tissues, particularly old leaves, suggesting that it may play a vital role under excess Fe conditions. However, the mechanism by which *OsNAS3* responds to excess Fe in rice remains poorly understood. In this study, we clarified the physiological response of *OsNAS3* expression to excess Fe and the role of NA synthesis in this condition. Promoter *GUS* analyses revealed that *OsNAS3* was widely expressed in roots, especially in vascular bundle, epidermis, exodermis, stem, and old leaf tissues under Fe excess compared to control plants. Nicotianamine and deoxymugineic acid (DMA; a type of phytosiderophore synthesized by Strategy II species) were present in roots and shoots under Fe excess likewise under control conditions. In addition, *OsNAS3* knockout plants were sensitive to excess Fe, exhibiting inferior growth, reduced dry weight, severer leaf bronzing, and greater Fe accumulation in their leaves than non-transformants with excess Fe. We also observed that NA-overproducing rice was tolerant of excess Fe. These results show that NA synthesized by *OsNAS3* under Fe excess condition is to mitigate excess Fe whereas NA synthesized by *OsNAS1* and *OsNAS2* under normal Fe condition is to enhance Fe translocation, suggesting the different roles and functions of the NA existence between these two conditions. Overall, these findings suggest that rice synthesizes NA with *OsNAS3* under Fe excess in roots and shoots, and that NA and DMA within the plant body are important for mitigating excess Fe stress and alleviating other metal deficiencies in rice. This report will be important for the development of tolerant rice adapted to Fe-contaminated soils.

## Introduction

Iron (Fe) is an essential nutrient for most living organisms and is a key determinant of crop production, yield, and quality; however, it can be toxic when hyperaccumulated within cells. Iron toxicity is one of the most important stressors of rice in many lowland environments worldwide. Iron is reduced from ferric ion [Fe(III)] to more soluble ferrous ion [Fe(II)] when submerged in water. Ferrous ion is more easily dissolved in water (*K*_sp_ = 8 × 10^-16^) compared to ferric ion (*K*_sp_ = ∼1 × 10^-36^) at 25°C (Stumm and Lee, 1961). Thus, Fe toxicity occurs more readily in submerged environments with acid soils. Acid soils occupy approximately 30% (or 3950 million hectares) of land worldwide, and approximately 50% of the world’s arable land is estimated to be acidic (von Uexküll and Mutert, 1995). Iron toxicity is a serious constraint on rice growth and yield in regions where rice production is high such as China and Southeast Asia, and the regions mainly characterized by acid soils such as ultisols (NRCS, 2005). Iron overload in plants leads to leaf bronzing, tissue damage, and cellular homeostasis disorders. To mitigate this problem and maintain Fe homeostasis within the plant body, plants use strict and sophisticated Fe regulation mechanisms.

Nicotianamine (NA) is a ubiquitous plant-derived chelator of various divalent cations such as Fe^2+^ and Zn^2+^, and is biosynthesized from S-adenosylmethionine by NA synthase (NAS; Higuchi et al., 1994). All higher plants synthesize NA and utilize it for chelation as well as internal transport of Fe^2+^ and other metal cations to maintain metal homeostasis (Hell and Stephan, 2003; Takahashi et al., 2003). Numerous studies have reported disrupted internal metal transport in NA-defective plants. For example, NA-defective tomato mutant exhibits an iron-deficient phenotype (Pich and Scholz, 1996; Stephan et al., 1996), NA-deficient transgenic tobacco plants have serious chlorosis in young leaves and decreased Fe and zinc (Zn) concentrations in leaves and flowers (Takahashi et al., 2003), and the *Arabidopsis AtNAS* quadruple mutant has a decreased Fe concentration in its seeds (Klatte et al., 2009). Introducing overexpression of a barley *NAS* gene, *HvNAS1*, to tobacco plants leads to increased Fe and Zn concentrations in the leaves, flowers, and seeds (Takahashi et al., 2003). These reports suggest that NA plays an important role in internal Fe transport in higher plants.

In rice, NA is biosynthesized by three NAS enzymes: *OsNAS1, OsNAS2*, and *OsNAS3* (Higuchi et al., 2001), all of which exhibit NA synthase activity *in vitro* (Inoue et al., 2003). Among three encoding genes, expression of *OsNAS1* and *OsNAS2* is strongly induced in rice roots and yellow leaves under Fe deficiency, whereas *OsNAS3* expression is mildly induced in roots but is suppressed in leaves under Fe deficiency (Inoue et al., 2003). The sequences of *OsNAS1* and *OsNAS2* are located very close to each other on rice chromosome 3, whereas *OsNAS3* is located on chromosome 7 (Higuchi et al., 2001; Inoue et al., 2003). Many other genes involved in Fe acquisition under Fe deficiency also show Fe deficiency-induced expression changes similar to those of the *OsNAS1* and *OsNAS2* genes, but not that of *OsNAS3* (Kobayashi et al., 2014). Thus, *OsNAS3* has a unique expression pattern compared to *OsNAS1* or *OsNAS2*, and as such, likely plays a different role than *OsNAS1* and *OsNAS2*.

Nicotianamine is not only involved in long-distance Fe transport in rice, but also serves as a substrate for production of deoxymugineic acid (DMA) via a 3″–oxo intermediate by NA aminotransferase (NAAT) and DMA synthase (DMAS) (Mori and Nishizawa, 1987; Shojima et al., 1989). In rice, one *NAAT* gene (*OsNAAT1*) and one *DMAS* gene (*OsDMAS1*) have been isolated (Bashir et al., 2006; Inoue et al., 2008). For DMA, it serves as a type of mugineic acid family phytosiderophore synthesized and secreted from graminaceous plants under Fe-deficient conditions to acquire Fe from the soil (Takagi, 1976; Marschner et al., 1986; Marschner and Romheld, 1994). Iron is thought to chelate with NA and DMA in the rice plant body and is translocated to various tissues including seeds. A correlation has been found between seed NA or DMA concentration and seed Fe concentration in transgenic rice with higher *NAS* expression (Masuda et al., 2009; Johnson et al., 2011).

Nicotianamine also plays a vital role in enhancing the nutritional quality of rice and is a candidate for biofortification of rice. For example, overexpression of the *NAS* gene increases Fe and Zn concentrations in rice seeds (Lee et al., 2009; Masuda et al., 2009; Johnson et al., 2011) and rice *glutelin B1* promoter-driven *OsNAS1* increases Fe concentrations in leaves and polished seeds (Zheng et al., 2010). Nicotianamine works synergistically with other genes to achieve high Fe in rice grains. For example, *HvNAS1, GmFER*, and *OsYSL2* (Masuda et al., 2012; Aung et al., 2013), *OsNAS2* and *GmFER* (Trijatmiko et al., 2016), and *AtNAS1, AtIRT1*, and *PvFER* (Boonyaves et al., 2017) together contribute to grain Fe accumulation. The increased Fe content in rice grains caused by enhanced *NAS* expression is also bioavailable (Lee et al., 2009). These reports suggest that NA synthesized by NAS enhances Fe translocation within rice plants to the seeds.

Nicotianamine is thought to play vital roles not only in Fe deficiency tolerance and seed Fe accumulation but also in detoxification of excess intracellular Fe (von Wirén et al., 1999). Former studies have shown the importance of NA in heavy metal metabolism in dicot plants (Pich et al., 2001; Takahashi et al., 2003; Douchkov et al., 2005; Kim et al., 2005; Han et al., 2018) and monocot plants (Lee et al., 2009; Aung et al., 2018). For example, among dicot plants, Pich et al. (2001) suggested a possible role of NA in vacuolar sequestration for detoxification of excess Fe in pea and tomato. In other dicots, transgenic *Arabidopsis* and tobacco plants overexpressing *HvNAS1* tolerated excess metal toxicity, particularly Ni (Kim et al., 2005). In addition, overexpression of apple *MxNAS1, MxNAS2* and *MxNAS3* genes led to enhanced tolerance to low and high levels of Fe stress in transgenic tomato (Han et al., 2013; Yang et al., 2015) and transgenic *Arabidopsis* by influencing NA synthesis (Han et al., 2018), respectively.

In dicot plants, NA functions exclusively as a metal chelator, as they do not produce MAs. In graminaceous plants, NA plays two roles, functioning not only as a metal chelator for internal transport but also as a precursor of MAs. In the monocot rice, overexpression of *OsNAS3* increased Fe and Zn concentrations and NA levels in grains as well as tolerance to Fe and Zn deficiencies and Zn, copper (Cu), and nickel (Ni) toxicities (Lee et al., 2009). Recently, we reported the transcriptomic analyses of various rice tissues in response to ferrous Fe toxicity (Aung et al., 2018). The results showed that Fe homeostasis-related genes were suppressed under excess Fe, principally in the roots. In particular, the NA synthase genes *OsNAS1* and *OsNAS2* were clearly suppressed in the roots and junction nodes between the root and shoot (discrimination center, DC). By contrast, *OsNAS3* expression was increased in all tissues in response to excess Fe, by 2- to 10-fold in the roots, 2- to 9-fold in the DC, 2- to 8-fold in stems, 10- to 120-fold in old leaves and 3- to 22-fold in the newest leaves. Under Fe excess, *OsNAS3* expression was as high as that of other important Fe excess-responsive genes, such as those of the Fe storage protein ferritin and vacuolar Fe transporter *OsVIT2* (Aung et al., 2018). These results suggest that NA synthesized by induced *OsNAS3* may play an important role in rice under Fe excess conditions. However, further in-depth analyses of *OsNAS3* are required to elucidate its physiological function in Fe excess and its role in Fe detoxification in rice.

In this study, we clarified the roles of NA and *OsNAS3* in rice under Fe excess conditions. To this end, we analyzed spatial expression patterns in various tissues of *OsNAS3* promoter-*GUS* plants (roots, DC, stems, old leaves, and newest leaves) in response to Fe excess. In addition, the amounts of NA and DMA in roots and shoots were investigated. An *OsNAS3* knockout line was cultivated under control and Fe excess conditions for observation of growth, leaf bronzing, metal concentrations, and Fe histochemical localization. Moreover, tolerance to Fe excess was observed in a NA-overproducing rice line. These results suggest a novel role for NA synthesized by *OsNAS3* in the Fe detoxification process.

## Materials and Methods

### Plant Materials and Growth Conditions in Hydroponic Culture

#### Plant Cultivation by Hydroponic Culture

Rice seeds were germinated on Murashige and Skoog (MS) medium (Murashige and Skoog, 1962) with and without hygromycin B (50 mg L^-1^) for transformant and non-transformant (NT) plants, respectively. The seedlings were grown in modified Kasugai’s hydroponic culture solution (× 1 Fe; 35.7 μM FeCl_2_) at pH 5.5 for 1 week, as previously described by Aung et al. (2018), after which the plants were cultivated under conditions of control Fe (× 1 Fe) or ferrous Fe excess (× 70 Fe; 2.50 mM FeCl_2_) at pH 4.0. The pH of the solution was adjusted to 4.0 every 2 days, and the culture solution was renewed every week. One plant per hill and three or four biological replicates were used for each Fe condition. All experiments were conducted in a greenhouse at 30°C during the 14 h day and 25°C during the 10 h night with natural light.

#### Growth Analyses of *OsNAS3* Knockout Rice Plants

Rice seeds (*Oryza sativa* L. cv. Hwayoung) of NT and the *OsNAS3* knockout mutant line 2D30228, *osnas3–1* (Lee et al., 2009) were obtained from the Rice T-DNA Insertion Sequence Database (POSTECH; Pohang University of Science and Technology, Pohang, Korea; Jeong et al., 2002). The T-DNA insertion position was described by Lee et al. (2009).

The 18-day-old seedlings (first experiment) or 14-day-old seedlings (second experiment) of knockout and NT plants were cultured hydroponically and exposed to excess Fe in hydroponic culture as described above for 39 days (first experiment) or 23 days (second experiment). Four biological replicates were used for each Fe condition. In both experiments, shoot and root lengths were measured throughout cultivation. The severity of Fe toxicity in leaves was measured after 11 or 17 days of exposure to excess Fe by determining the bronzing score of the fully expanded newest leaf (NL) and older leaves, as previously described by Aung et al. (2018).

#### Growth Analyses of NAS-Overexpressing Rice Plants

Rice seeds (*O. sativa* L. cv. Tsukinohikari) of NT plants and the T_4_ generation of NAS-overexpressing (*Actin* promoter-*HvNAS1*) plants (Masuda et al., 2009) were used for growth analyses. Eighteen-day-old seedlings were cultured hydroponically and exposed to excess Fe in hydroponic culture for 39 days. Plant growth and bronzing scores were measured as described above.

### Histochemical Analyses of Promoter-GUS Rice Lines

The seeds of the *OsNAS3* promoter-*GUS* lines (T_3_ and T_4_ seeds; *O. sativa* L. cv. Tsukinohikari) described by Inoue et al. (2003) were used for histochemical analyses. Fifteen-day-old seedlings were transferred to hydroponic culture and exposed to excess Fe for 14 days. Roots, leaf blades, stems, DCs were cut with a scalpel into approximately 1 cm sections. Then, the sections were embedded into 5% agar and cut into 100 μm transverse, or longitudinal sections by a DTK-1000N MicroSlicer (Dosaka EM Co., Ltd., Kyoto, Japan) as described by Kobayashi et al. (2010). GUS reaction buffer was prepared with 1 mM 5-bromo-4-chloro-3-indolyl-ß-D-glucuronide Cyclohexyl ammonium salt (X-Gluc; Wako, Japan) in staining buffer (100 mM Na_2_HPO_4_, 1 mM K_3_Fe(CN)_6_, 1 mM K_4_Fe(CN)_6_, and 20% methanol). The samples were subjected to histochemical assays for GUS activity, with vacuum infiltration on ice for 30 min and then incubated at 37°C dark for 7 min for roots, 1 min for DCs, 9 min for stems, 2 h 30 min for old leaves and 1 h for the newest leaves. Once the staining appeared, the reactions of both control and Fe excess sections were stopped at the same time by washing 70% ethanol and then reserved in 70% ethanol. Histochemical localization was observed in both the T_3_ and T_4_ generations of at least two independent lines under a Zeiss Axion Vision Image 4.2 microscope and a Zeiss Axioskop 2 Plus fluorescence microscope (Carl Zeiss Microscopy, Jena, Germany).

### NA and DMA Concentration Analyses

Rice seeds (*O. sativa* L. cv. Tsukinohikari) were used for NA and DMA concentration analyses. Sixteen-day-old rice seedlings were transferred to hydroponic culture, and half were exposed to excess Fe at pH 4.0 for 14 days. Four biological replicates were used in control (× 1 Fe) and Fe excess (× 70 Fe) hydroponic cultures. Leaf and root samples were ground to powder in liquid nitrogen using a mortar and pestle, and then the endogenous NA and DMA concentrations of the samples were determined through high-performance liquid chromatography according to the method described by Nozoye et al. (2014).

### Metal Concentration Analyses

Samples of roots and shoots (newest leaves and third newest leaves) from control and Fe-treated plants (from the second experiment; 23 days of excess Fe exposure) were collected for metal concentration analyses. The roots were washed with distilled water or Milli-Q water containing 50 mM sodium ethylenediaminetetraacetic acid (Na-EDTA) for control or Fe excess plants, respectively. Root and leaf samples were dried for 3 days at 60°C, and 50–200 mg samples were digested with 2 mL HNO_3_ and 2 mL H_2_O_2_ at 220°C for 20 min using the MARS XPRESS oven (CEM Japan, Tokyo, Japan), and then messed up and filtered as described by Masuda et al. (2009). The concentrations of Fe, Zn, manganese (Mn), and Cu were measured with an inductively coupled plasma atomic emission spectrometer (ICPS-8100; Shimadzu, Kyoto, Japan). Three biological replicates were performed.

### Iron Histochemical Localization Analyses

To detect the presence of Fe in the leaf tissues of *OsNAS3* knockout plants, leaf sections from control and Fe excess cultures were obtained (from the second experiment) and placed in ethanol for 24 h to remove the chlorophyll. Then the sections were exposed to a solution containing 2% potassium ferricyanide (Wako Co., Ltd., Tokyo, Japan) and 2% hydrochloric acid (Wako) for 24 h. After rinsing with distilled water, the sections were mounted in distilled water and localization was observed under the Zeiss Axion Vision Image 4.2 microscope (Carl Zeiss Microscopy).

### Quantitative Real-Time RT-PCR Analyses

Total RNA from rice prepared for the microarray analyses as described by Aung et al. (2018) was used to confirm the expression patterns of the genes *OsDMAS1* and *OsNAAT1* under Fe excess conditions by quantitative real-time polymerase chain reaction (RT-PCR) analyses. For the expression analyses of *OsNAS3* knockout and NT plants from ×1 Fe and ×70 Fe cultures, rice RNA was extracted from hydroponically grown leaves. For all samples, the first-strand cDNA was synthesized using the ReverTra Ace reverse transcriptase kit (Toyobo, Osaka, Japan) and oligo-d(T)_30_ primers. Then, qPCR was performed in a StepOnePlus^TM^ Real-Time PCR System (Life Technology, Tokyo, Japan) with SYBR Premix Ex Taq II reagent (Takara, Shiga, Japan). The transcript abundance was normalized against the rice *alpha-tubulin* transcript level. The primer sequences used for gene expression analyses were as follows: 5′GCC GGC ATC CCG GCA GCG GAA GAT CA 3′ for *OsDMAS1* FW and 5′ CTC TCT CTC TCG GGC ACG TGC TAG CGT 3′ for *OsDMAS1* RV; 5′-TAAGAGGATAATTGATTTGCTTAC-3′ for *OsNAAT1* FW and 5′-CTGATCATTCCAATCCTAGTACAAT-3′ for *OsNAAT1* RV; 5′ CGA TGA CTG CTT CCA TCG CTT G 3′ for *OsNAS3* FW and 5′ GGC A TG CAT TCA TGC ATG ACT GC 3′ for *OsNAS3* RV; 5′ TCT TCC ACC CTG AGC AGC TC 3′ for *alpha-tubulin* FW and 5′ AAC CTT GGA GAC CAG TGC AG 3′ for *alpha-tubulin* RV.

### Statistical Analyses

Statistical analyses were conducted using Microsoft Excel software. Comparisons were made between ×1 Fe and each level of Fe excess in each tissue. For each set of comparisons, a two-sample Student’s *t*-test for an equal or unequal variance was performed based on the *F*-test for equal variance.

## Results

### Expression of *OsNAS3* Was Observed in All Tissues Investigated Under Fe Excess

**FIGURE 1:**
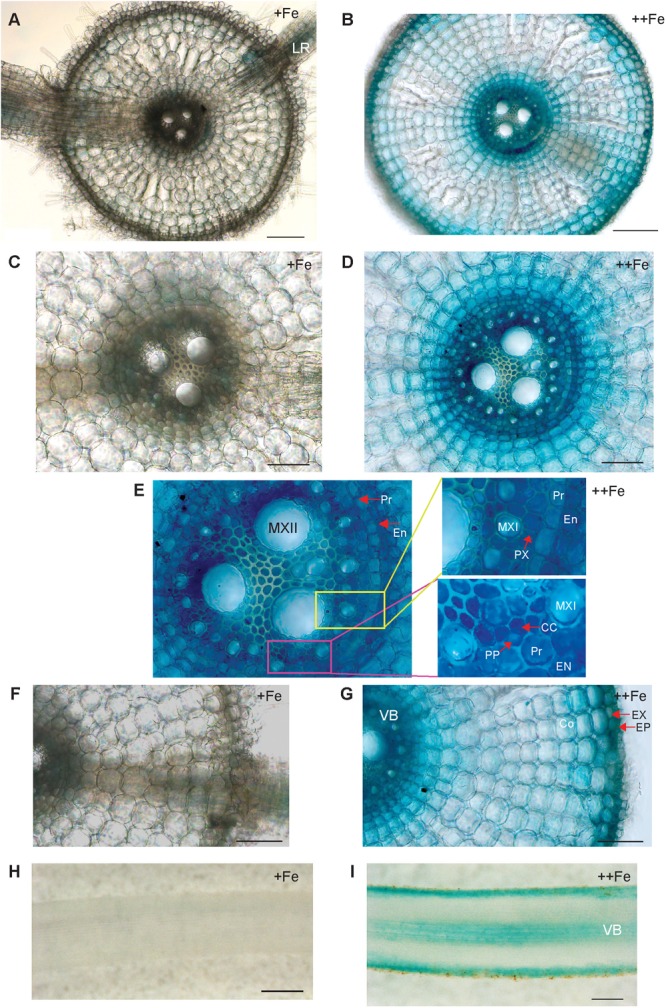
Cellular localization of *OsNAS3* promoter-GUS expression in roots. **(A)** Transverse section of control root. **(B)** Transverse section of Fe-excess root. **(C,D)** Enlarged views of vascular tissues shown in **(A,B)**, respectively. **(E)** An enlarged view of xylem and phloem shown in **(D)**. **(F,G)** Enlarged views of outer root layers shown in **(A,B)**, respectively. **(H)** Longitudinal section of control root. **(I)** Longitudinal section of Fe-excess root. LR, lateral root; MXI, metaxylem I; MXII, metaxylem II; PP, protophloem; CC, companion cells; EN, endodermis; Pr, pericycle; PX, protoxylem; EX, exodermis; EP, epidermis; Co, cortex; VB, vascular bundle. Scale bars: 20 μm for **(A–D,F,G)**; 100 μm for **(H,I)**.

To uncover the physiological role of *OsNAS3* in Fe excess, the tissue-specific expression of *OsNAS3* was investigated based on histochemical localization in transgenic rice plants with the introduced *OsNAS3* promoter–*GUS* described by Inoue et al. (2003). Expression of *OsNAS3* was observed in every tissue investigated. In the transverse section of roots under control Fe conditions, *OsNAS3* expression was mainly restricted to phloem cells and lateral roots (Figure 1A,C,F). Under excess Fe, strong expression was observed throughout the entire root, particularly in the vascular bundles, exodermis, and epidermis of the roots observed in transverse sections (Figure 1B,D,E,G). Enlarged transverse sections of the vascular bundle exhibited extremely deep staining in the phloem cells, phloem companion cells, protoxylem, xylem parenchyma cells, and epidermal cells (Figure 1D,E). In the longitudinal sections, *OsNAS3* expression in roots was very clear under Fe excess compared to control conditions (Figure 1H,I). Expression of *OsNAS3* was observed in lateral roots in rice under both control and Fe excess conditions (Figure 1A and Supplementary Figure S1A).

**FIGURE 2:**
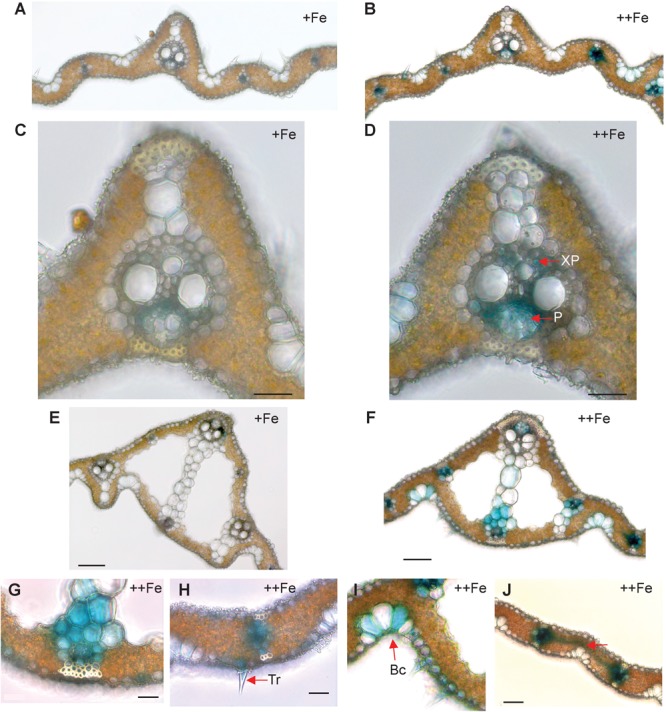
Cellular localization of *OsNAS3* promoter-GUS expression in old leaves. **(A)** Transverse sections of control old leaf. **(B)** Fe-excess old leaf. **(C)** Enlarged view of control leaf. **(D)** Enlarged view of Fe-excess leaf. **(E)** Midrib of control leaf. **(F)** Midrib of Fe-excess leaf. **(G)** An enlarged view of **(F)**. **(H)** Fe excess leaf showing trichome. **(I)** Fe excess leaf showing bulliform cells. **(J)** Vascular vein connecting vascular bundles of Fe-excess leaf shown by arrow. XP, xylem parenchyma; P, phloem; Tr, trichome; Bc, bulliform cells. Scale bars: 20 μm for **(C–J)**.

**FIGURE 3:**
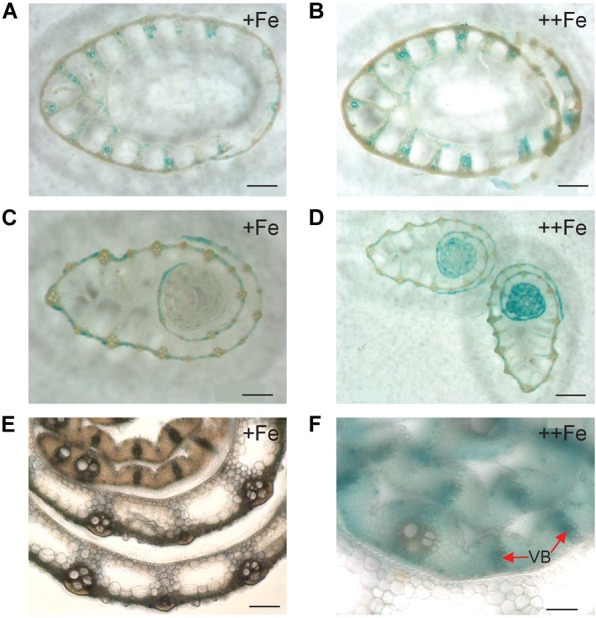
Cellular localization of *OsNAS3* promoter-GUS expression in the stem. **(A)** The outer part of the control stem. **(B)** The outer part of the Fe-excess stem. **(C)** The inner part of the control stem. **(D)** The inner part of the Fe-excess stem. **(E,F)** Enlarged views of **(C,D)**, respectively. VB, vascular bundle. Scale bars: 500 μm for **(A–D)**; 20 μm for **(E–F)**.

In old leaf blades, weak expression of *OsNAS3* under Fe sufficiency was present only in the small vascular bundles and phloem cells of large vascular bundles (Figure 2A,C). Under Fe excess, leaves showed stronger expression of *OsNAS3* in the small and large vascular bundles (Figure 2B) and xylem parenchyma and phloem cells (Figure 2D). In addition, weak expression was observed in extracellular veins and chloroplasts within mesophyll cells in leaves with excess Fe (Figure 2D and Supplementary Figure S1B). In the midrib of the control Fe leaf, expression was very weak (Figure 2E), whereas the Fe excess leaf midrib showed dominant expression of *OsNAS3* in the vascular bundles, bundle sheath cells and adjacent cells, the lower and upper epidermis (Figure 2F,G), trichomes (Figure 2H), some parts of the bulliform (motor) cells present on the adaxial side of the leaf (Figure 2I), and also in the veins connecting small vascular bundles (Figure 2J). The outer layer of the stem showed dominant expression under excess Fe than under control (Figure 3A,B). In the inner part of the stem, expression of *OsNAS3* in the prospective new leaves that would soon emerge, but were folded inside stem tissue at the time, was more pronounced under excess Fe compared to control Fe condition (Figure 3C–F).

**FIGURE 4:**
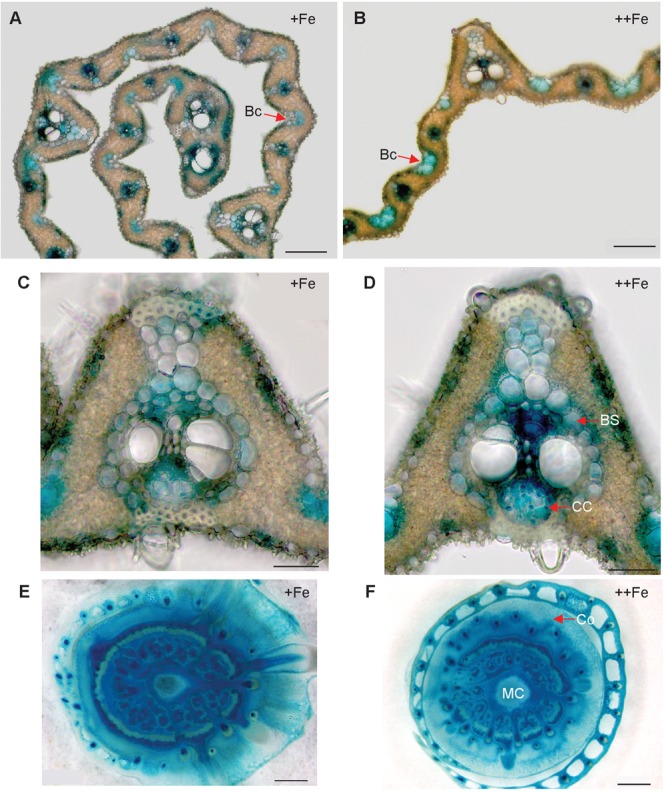
Cellular localization of *OsNAS3* promoter-GUS expression in the newest leaves and DCs. **(A)** Transverse sections of the control newest leaf. **(B)** Fe-excess newest leaf. **(C,D)** Enlarged views of **(A,B)**, respectively. **(E)** Control DC. **(F)** Fe-excess DC. Bc, bulliform cells; BS, bundle sheath cells; CC, companion cells; Co, cortex; MC, medullary cavity. Scale bars: 20 μm for **(A–D)**; 500 μm for **(E,F)**.

In the newest leaves, *OsNAS3* expression patterns were not affected by Fe status (Figure 4A–D). Expression was localized to the small and large vascular bundles, bundle sheath cells, some parts of mesophyll cells and collenchyma fibers, and bulliform and epidermis cells of both the abaxial and adaxial surfaces (Figure 4A–D and Supplementary Figures S1C,D). More dense and dominant GUS activity was observed inside the large vascular bundles of leaves particularly in phloem companion cells under Fe excess than in control leaves. Bulliform cells, which only expressed *OsNAS3* under Fe excess conditions in old leaves, expressed *OsNAS3* in the newest leaves under both control and Fe excess conditions (Figure 4A,B). Bundle sheath cells, which did not express *OsNAS3* in old leaves, expressed this gene dominantly in the newest leaves (Figure 4C,D). DCs constitutively exhibited strong *OsNAS3* expression, particularly in the thick cortex, medullary cavity, and leaf sheath of the second leaf, whether Fe was sufficient or excessive (Figure 4E,F and Supplementary Figures S1E,F). Expression patterns were similar for DCs in the control and Fe excess conditions.

### Expression of *OsDMAS1* in Various Rice Tissues

**FIGURE 5:**
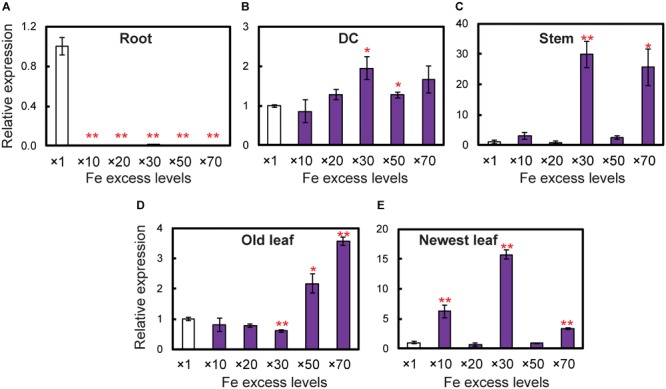
Expression levels of the *OsDMAS1* gene in rice tissues under control and various Fe excess conditions. **(A)** Roots. **(B)** DCs. **(C)** Stems. **(D)** Old leaves. **(E)** Newest leaves. DC, discrimination center. This figure shows confirmation of the microarray results listed in Table 1 of Aung et al. (2018) from qPCR analyses. Error bars represent ± 1 standard error (SE) of the technical variation, *n* = 3. Data were normalized to the observed expression levels of *alpha-tubulin* and presented as relative gene expression in each tissue (×1 Fe = 1). Asterisks above the bars indicate significant differences (^∗^*P* < 0.05; ^∗∗^*P* < 0.01) compared to the control (×1 Fe).

To determine whether *OsDMAS1* and *OsNAAT1* are involved in DMA synthesis in rice exposed to excess Fe, we analyzed the expression patterns of these genes in various tissues (Figure 5). In roots, *OsDMAS1* expression was reduced but not completely suppressed under Fe excess compared to control conditions (Figure 5A). In aboveground tissues (i.e., DCs, stems, old leaves and newest leaves), the expression of *OsDMAS1* was similar under both control and Fe excess conditions, or higher under excess Fe conditions (Figure 5B–E). We also measured *OsNAAT1* expression, which was reduced in roots, but not completely suppressed. Its levels remained similar or increased with Fe excess in DC, old leaf and newest leaf tissues (Supplementary Figure S2).

### Endogenous NA and DMA Are Present in Both Roots and Shoots With Excess Fe

**FIGURE 6:**
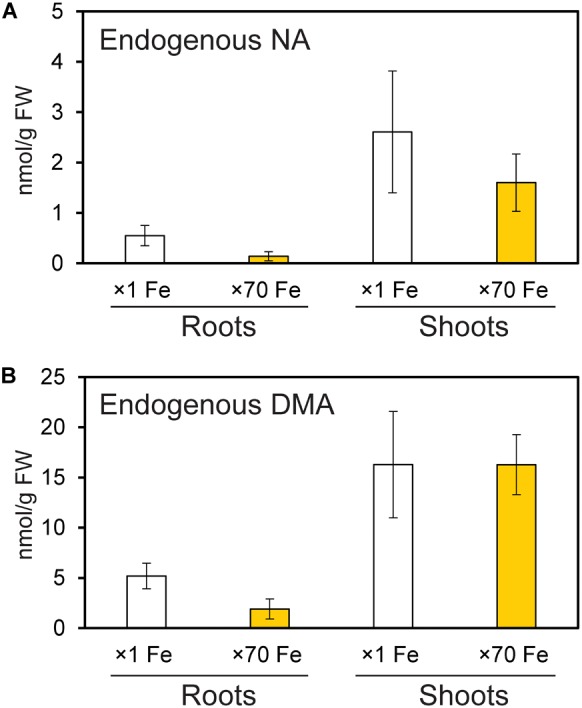
Amounts of endogenous nicotianamine (NA) and deoxymugineic acid (DMA) in the roots and shoots of control and Fe-excess rice. **(A)** The amount of endogenous NA and **(B)** the amount of endogenous DMA in roots and shoots of non-transformant rice under control and Fe excess conditions. Plants were grown hydroponically under control (×1 Fe) and excess ferrous Fe (×70 Fe) conditions at pH 4.0 for 14 days. Error bars represent ± 1 SE of three biological replicates. There were no significant differences between × 1 Fe and × 70 Fe in roots or shoots in **(A,B)**, as determined using a *t*-test (^∗^*P* < 0.05; ^∗∗^*P* < 0.01).

Endogenous NA and DMA concentrations were analyzed in roots and shoots of NT rice grown under control and Fe excess conditions, and were present in both roots and shoots under excess Fe and control Fe conditions (Figure 6). Shoots had higher concentrations of NA and DMA than roots, and these concentrations did not significantly differ between control and Fe excess plants.

### The *OsNAS3* Knockout Plants Are Weaker Under Excess Fe

**FIGURE 7:**
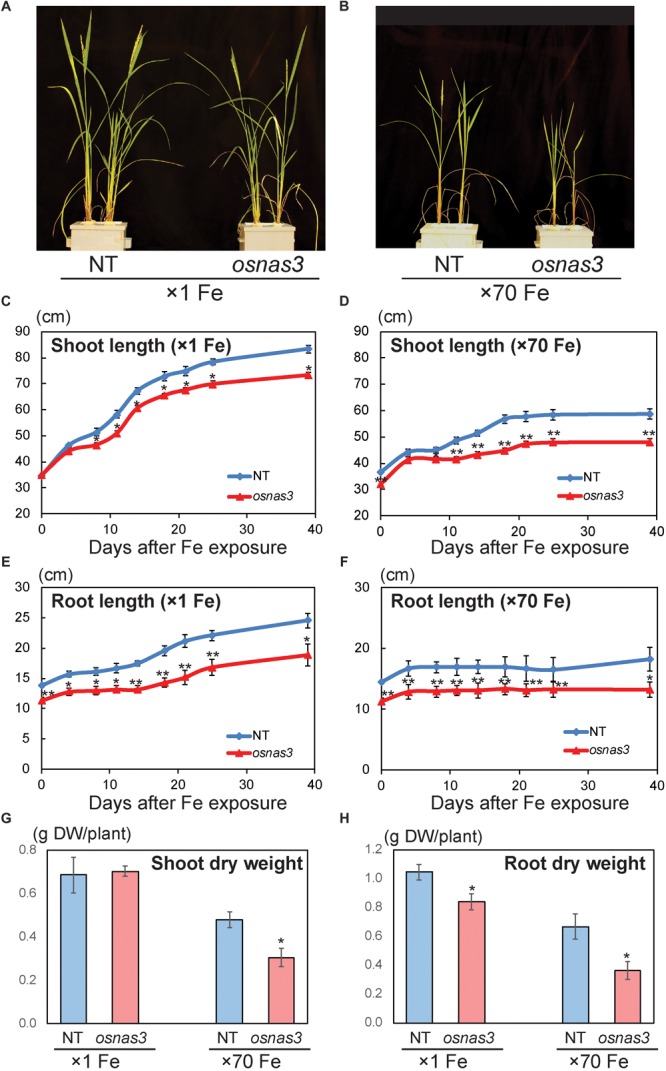
Plant appearance and growth of NT and *OsNAS3* knockout rice. **(A,B)** Appearance of *OsNAS3* knockout plants and non-transformants (NT) under control **(A)** or Fe excess **(B)** conditions after 39-day treatment. **(C,D)** Shoot length under control **(C)** or Fe excess **(D)** conditions. **(E,F)** Root length under control **(E)** or Fe excess **(F)** conditions. **(G,H)** Dry weights of shoots **(G)** and roots **(H)** under control and Fe excess condition at 23 days after transplanting (from 2nd experiment). Error bars represent the standard error (SE) of biological replicates, *n* = 4 for NT and *n* = 3 for knockout plants. Plants were grown hydroponically under control (×1 Fe) or excess ferrous Fe (×70 Fe) conditions at pH 4.0. Asterisks indicate significant differences compared to the NT at each time point or each Fe condition (^∗^*P* < 0.05, ^∗∗^*P* < 0.01).

**FIGURE 8:**
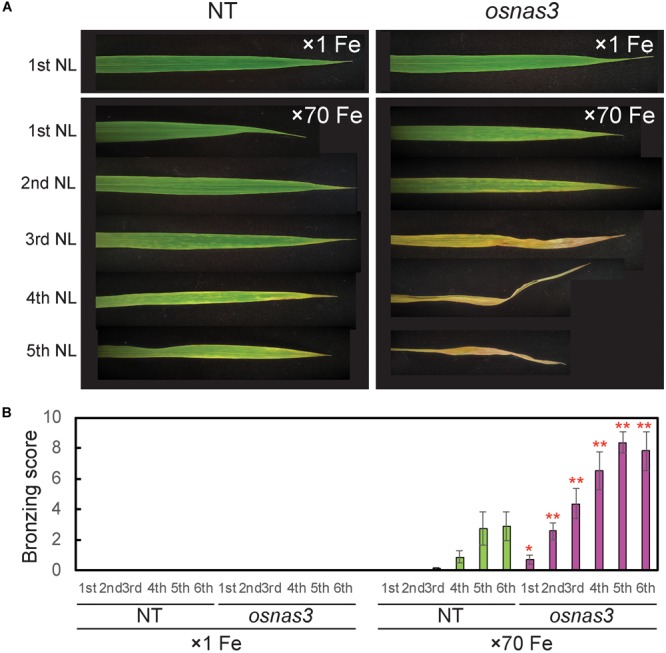
Leaf appearance and leaf bronzing scores of NT and *OsNAS3* knockout rice. **(A)** Bronzing symptoms on representative leaves after 16 days of control or excess Fe stress treatment. The first, second, third, fourth, and fifth newest leaves (NL) are shown. **(B)** Bronzing scores after 17 days of Fe exposure. The first, second, third, fourth, fifth and six newest leaves are indicated on the horizontal axis as 1st, 2nd, 3rd, 4th, 5th, and 6th, respectively. Error bars represent the SE of biological replicates, *n* = 8. Plants were grown hydroponically under control (×1 Fe) and excess ferrous Fe (×70 Fe) conditions at pH 4.0. Asterisks indicate significant differences compared to the NT for each Fe condition and plant part (^∗^*P* < 0.05, ^∗∗^*P* < 0.01).

The *OsNAS3* knockout mutant line described by Lee et al. (2009) was used to investigate the response to Fe excess. The knockout plants and NT were grown in hydroponic cultures with control and excess Fe conditions. We also measured *OsNAS3* expression in knockout plants under control and excess Fe conditions (Supplementary Figure S3). Under excess Fe supply, all knockout plants showed growth defects compared to NT plants (Figure 7A,B and Supplementary Figure S4). The NT plants under Fe-excess set panicles likewise under control Fe conditions but the knockout plants under Fe excess did not (Figure 7A,B). Shoot and root growth were significantly suppressed in knockout plants compared to NT under both control and excess Fe conditions (Figure 7C,D). Continuous growth was observed in both shoots and roots of all plants under control conditions (Figure 7C,E). Inferior shoot growth became pronounced in knockout plants with excess Fe after 7 days, and growth was stunted after 27 days, while root growth retardation occurred earlier, at 5 days after excess Fe exposure (Figure 7D,F). In the second experiment, the dry weight of roots and shoots were also measured. The shoot dry weights of the knockout plants were similar with NT under control condition, but reduced 36% in excess Fe compared with NT (Figure 7G). The root dry weights of the knockout plants reduced 20% in control condition and 46% in Fe excess condition compared to those of NT (Figure 7H). In this experiment, rather than the growth defect, the decrease in dry weight and the leaf bronzing caused by Fe excess damage in knockout plants were more pronounced. Bronzing symptoms on leaves were observed and bronzing scores were recorded for control and Fe-excess NT and *OsNAS3* knockout plants. All leaves were healthy in both NT and knockout plants under control Fe conditions (Figure 8A). By contrast, *OsNAS3* knockout plants showed serious leaf bronzing, particularly in older leaves, with excess Fe compared to NT plants (Figure 8A). Higher bronzing scores in all leaves of knockout plants (first to sixth newest leaves) were observed compared to those of NT plants exposed to excess Fe (Figure 8B).

**FIGURE 9:**
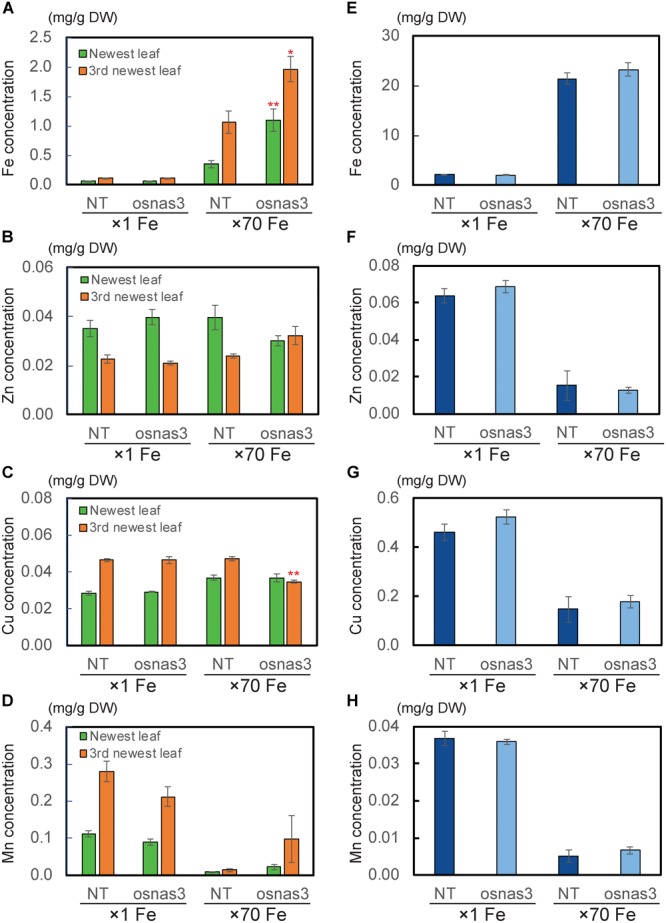
The metal concentrations in NT and *OsNAS3* knockout plants under control and Fe excess conditions. **(A)** Fe, **(B)** Zn, **(C)** Cu and **(D)** Mn concentrations in leaves. **(E)** Fe, **(F)** Zn, **(G)** Cu and **(H)** Mn concentrations in roots. Plants were grown hydroponically under control (×1 Fe) and excess ferrous Fe (×70 Fe) conditions at pH 4.0 for 23 days. Green and orange bars indicate concentrations in the newest and third newest leaves, respectively. Error bars represent ± 1 SE from 3 biological replicates. Asterisks above the bars indicate significant differences compared to NT for each condition and plant part (^∗^*P* < 0.05; ^∗∗^*P* < 0.01).

**FIGURE 10:**
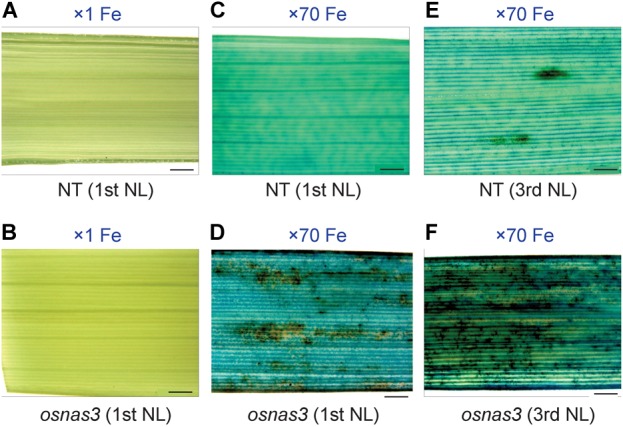
Histochemical Fe localization in leaves of NT and *OsNAS3* knockout rice determined through Prussian blue staining. **(A)** First newest leaf of NT and **(B)** first newest leaf of knockout plant under control Fe conditions. **(C)** First newest leaf of NT, **(D)** first newest leaf of knockout plant, **(E)** third newest leaf of NT and **(F)** third newest leaf of knockout plant under Fe excess conditions. NL, newest leaf. Plants were grown hydroponically under control (×1 Fe) and excess ferrous Fe (× 70 Fe) conditions at pH 4.0 for 23 days. Scale bars: 500 μm.

We also measured metal concentrations in the newest leaves, third newest leaves (older leaves), and whole roots of *OsNAS3* knockout mutants and NT plants grown for 23 days under either control or Fe excess conditions (Figure 9). With excess Fe, both NT and knockout plants showed higher Fe accumulation in the newest leaves, which was even higher in older leaves, compared to the control condition (Figure 9A). In particular, *OsNAS3* knockout plants had significantly higher Fe accumulation in both the newest (3 times higher) and older leaves (2 times higher) compared to the NT under excess Fe, while there were no differences from the NT in the control Fe treatment (Figure 9A). The Zn and Cu concentration trends did not differ between knockouts and NT under control conditions, but these trends were altered with excess Fe (Figure 9B,C). Both NT and knockout plants had higher Zn and lower Cu concentrations in the newest leaves than in older leaves. The concentrations of these metals were similar between the newest and older leaves of knockout plants with excess Fe (Figure 9B,C). Under excess Fe, knockout plants tended to have lower Zn in the newest leaves and lower Cu in older leaves compared to NT (Figure 9B,C). Mn accumulation was greater in older leaves than the newest leaves in the control condition, and its accumulation decreased with excess Fe in both NT and knockout plants (Figure 9D). Under excess Fe conditions, knockout plants tended to have higher Mn accumulation than NT plants, particularly in older leaves. The concentrations of Fe in roots were elevated in both knockouts and NT plants grown under excess Fe compared to control treatments, but there were no significant differences between knockout mutants and NT plants (Figure 9E). In roots, plants treated with excess Fe had lower concentrations of Zn, Cu, and Mn (Figure 9F–H). These metal accumulations were unaltered between NT and knockout plants, regardless of Fe status (Figure 9F–H). To evaluate how Fe is localized in leaf tissues of knockout plants, we observed Fe histochemical localization as deep blue staining using the Prussian blue staining method. Under control Fe conditions, both NT and knockout plants showed low Fe contents in leaves (Figure 10A,B). Iron staining was observed with Fe excess in both first and third newest leaves of NT plants (Figure 10C,E). Iron staining was more prominent in the newest leaves and in older leaves of Fe-excess knockout plants that exhibited serious leaf bronzing than in NT leaves (Figure 10D,F).

### Transgenic Line With Elevated NA Production Was More Tolerant to Excess Fe

To further examine the role of NA in the response to excess Fe in rice, a transgenic rice line with 15-fold elevated NA production in shoot tissues via induced expression of barley *HvNAS1* (Masuda et al., 2009) was cultivated under both control and Fe excess conditions, and its growth and morphological characteristics were observed. Under excess Fe, the high-NA line maintained its growth and remained healthy throughout cultivation for 39 days, showing increased tolerance to excess Fe compared to NT plants, and similar growth to NT plants under control conditions (Supplementary Figure S5).

## Discussion

### Expression of *OsNAS3* Is Investigated in Various Tissues Under Fe Excess Conditions

The localization of *OsNAS3* expression was investigated using promoter*-GUS* analyses to determine the physiological role of this gene under Fe excess conditions. The strong expression of *OsNAS3* was observed throughout Fe-excess root cells compared to control roots, suggesting that *OsNAS3* functions deep within roots in the presence of excess Fe. Inoue et al. (2003) showed that *OsNAS3* expression was slightly induced in roots under Fe deficiency. Thus, it is conceivable that *OsNAS3* preferentially works under stress conditions: it may function in roots weakly under Fe deficiency and strongly under excess Fe conditions.

Under excess Fe, *OsNAS3* expression was particularly dominant in exodermis, epidermis and vascular bundles (Figure 1), especially, extremely strong activity was observed in phloem cells, phloem companion cells, protoxylem, xylem parenchyma cells, and epidermal cells (Figure 1D,E). These results suggest that NA synthesis due to *OsNAS3* may be involved in the radial movement of Fe to vascular bundle cells, as well as chelating excess Fe in roots and enhancing xylem and phloem loading.

Inoue et al. (2003) reported that under Fe deficiency, *OsNAS3* expression was restricted to the central cylinder cells and did not extend to all root cells, suggesting that unlike *OsNAS1* and *OsNAS2, OsNAS3* does not contribute to the enhanced secretion of DMA from Fe-deficient roots. However, DMA accumulation was detected in the xylem sap of Fe-sufficient rice and under control Fe conditions (Kawai et al., 2001; Kakei et al., 2009). Our results also showed DMA accumulation in both control and Fe excess roots (Figure 5, 6). Expression of *OsNAAT1* and *OsDMS1* was observed in both control and excess Fe roots (Figure 5A and Supplementary Figure S2A), suggesting that NA is further converted into DMA in these cells under control and excess Fe conditions and that not only NA but also DMA may participate in Fe detoxification.

Stronger expression of *OsNAS3* was observed in Fe-excess old leaves compared to control Fe leaves (Figure 2). Under control Fe conditions, *OsNAS3* expression was restricted to phloem cells inside the large and small vascular bundles of old leaves (Figure 2A,C,E). Strong induction of *OsNAS3* expression in old leaves was also observed through microarray and qPCR analyses (Aung et al., 2018). The GUS analyses showed dominant expression in vascular bundles, but only weak expression throughout the leaves, particularly in mesophyll cells. This result might be due to Fe stress causing old leaves to turn bronze in color, associated with cell death due to Fe overload (Figure 2D,G–J and Supplementary Figure S1B). In this study, we applied strong Fe excess stress to plants (70 times the control level). Thus, an experiment using milder Fe excess stress may be more suitable for identifying differences in old leaves.

Expression of *OsNAS3* was observed in the trichomes of old leaves under excess Fe (Figure 2H). The Fe toxicity induces oxidative stress, which in turn causes limitation of photosynthesis. A rice variety that is sensitive to Fe excess showed impairment in light energy partitioning and oxidative damage before the onset of visual symptoms (Pinto et al., 2016). Some plant species accumulate excess metals in trichomes, cuticles or epidermal common cells to avoid greater damage to photosynthetic machinery (Küpper et al., 2000; Robinson et al., 2003; Freeman et al., 2006). Presumably, NA synthesis or allocation in trichomes and epidermal cells may be responsible for protecting leaf photosynthesis during Fe excess stress. Trichomes and epidermal cells showed high levels of GUS activity in the newest leaves irrespective of Fe status in this study (data not shown). *OsNAS3* expression was also frequently seen in bulliform (motor) cells in old leaves treated with excess Fe (Figure 2I). Bulliform cells act as an entrance for light into the mesophyll cells (Clayton and Renvoize, 1986) and participate in the folding of mature leaves to minimize water loss during drought as well as the expansion of young leaves rolled in the apex (Shields, 1951; Jane and Chiang, 1991). Similar *OsNAS3* expression in bulliform cells was observed in Fe-deficient leaves, suggesting a possible role of metals bound to NA in the regulation of water volume in bulliform cells (Inoue et al., 2003). Our result provides further support for *OsNAS3* expression in these cells being for young leaf expansion and protection against water loss induced by excess Fe stress.

Expression of *OsNAS3* was observed in both vascular bundles and mesophyll cells in the newest green leaves of both Fe sufficient and excess conditions (Figure 4C,D and Supplementary Figures S1C,D). Inoue et al. (2003) showed GUS expression in Fe sufficient green leaf tissue. Nicotianamine produced by *OsNAS3* in the newest leaves may chelate excess Fe to mitigate Fe excess damage to the newest leaves. Iron excess causes a deficiency of other metals, such as Zn (Aung et al., 2018). Thus, NA is thought to support to the transport of other minerals, including Zn, in these leaves. In the inner part of the stem, prospective new leaves folded inside stem tissues showed denser GUS staining under Fe excess conditions compared to the control (Figure 3C–F). These very young new leaves inside the inner part of the stem are prone to Fe exposure transported via phloem pathway, but less Fe exposure than the previously extended leaves where Fe is transported via xylem stream from Fe excess roots. Still, protection from Fe excess is thought to be very important for new leaves inside the stem. *OsNAS3* is thought to work in the next set of leaves to emerge when they are folded inside the stem. Discrimination center (DC) is a region that includes the shoot meristem, node, and internode (Mori, 1998; Itoh et al., 2005) and plays an important role in mineral and metabolite transport in graminaceous plants. Iron and other minerals absorbed by the roots first accumulate in the DC and then are distributed to shoots (Tsukamoto et al., 2009). *OsNAS3* is strongly induced in DC under Fe excess (Aung et al., 2018). In this study, the strong expression of *OsNAS3* was localized in DC, regardless of Fe status (Figure 4E,F and Supplementary Figures S1E,F). Iron chelated with NA controlled by *OsNAS3* in DC might be transported to the shoots through YSL transporters such as *OsYSL17* and *OsYSL18*, which are induced under Fe excess in the roots and DC (Aung et al., 2018).

### Endogenous NA and DMA Are Present in Both Roots and Shoots Under Excess Fe

Under both normal and limited Fe conditions, graminaceous plants produce NA, which is further converted into DMA to acquire Fe from the rhizosphere (Takagi, 1976; Marschner et al., 1986). Nicotianamine enhances Fe translocation within plants under both limited and normal Fe conditions (Takahashi et al., 2003). Our results confirmed the presence of NA in control roots (Figure 6). This NA was produced by *OsNAS1* and *OsNAS2* to enhance Fe translocation under normal condition. On the other hand, under Fe excess condition, plants might not need to produce further NA within the plant to take up more Fe. However, NA and DMA were present in roots and shoots under Fe excess (Figure 6). *OsNAS3* was strongly induced in most rice tissues under Fe excess compared to the control (Aung et al., 2018). Thus, this NA might be produced by *OsNAS3*. Under normal Fe condition (x 1 Fe), rice plants should have efforts to take up and translocate Fe within the plant body. Thus, the *OsNAS1* and *OsNAS2* expression was high in roots under x1 Fe condition for the transport of Fe and other metals within the plant body, whereas their expression in roots was extremely low under ×70 Fe condition (Aung et al., 2018). Moreover, the produced NA in roots will be transported to shoot under normal Fe condition together with other metal transportation. Because of this reason, although NA productivity increased by *OsNAS3* under Fe excess, total NA concentrations was the same or slightly reduced under Fe excess condition (Figure 6). These results clearly show that the roles and the functions of the NA between these two conditions are different. It is well understood in plant nutrition that NA is used for Fe translocation or a precursor of DMA for Fe uptake. Our finding is that NA and DMA still exist under Fe excess and there is a role of them under Fe excess. The expression of *OsNAAT1* and *OsDMAS1* in roots was reduced but not completely suppressed with excess Fe (Figure 5A and Supplementary Figure S2). Their expression levels were even higher under Fe excess in the DC, stem, old leaves, and newest leaves (Figure 5B–E and Supplementary Figure S2; Aung et al., 2018). Thus, DMA may also participate in Fe detoxification processes under Fe excess conditions. Free Fe causes cellular Fe toxicity because it serves as a catalyst in the formation of free radicals from reactive oxygen species via the Fenton reaction. But Fe chelated with NA does not undergo the Fenton reaction and can be safely sequestered and stored in ferritins or vacuoles within plant tissues. The Fe(II)-NA complexes are poor Fenton reagents, based on their ability to mediate H_2_O_2_-dependent oxidation of deoxyribose, suggesting that NA has a vital role in scavenging Fe and protecting the cell from Fe-induced oxidative damage (von Wirén et al., 1999). Thus, NA and DMA production by *OsNAS3* under Fe excess conditions is to chelate free excess Fe and thus mitigate Fe excess stress.

Among NAS genes in rice, *OsNAS3* belongs to Clade II, (Mizuno et al., 2003; Bonneau et al., 2016). Bonneau et al. (2016) reported that maize *ZmNAS3, ZmNAS4, ZmNAS5*, barley *NASHOR2*, wheat *TaNAS9-A, TaNAS9-B*, and *TaNAS9-D* are included in Clade II, and among these, wheat *TaNAS9-A* and *TaNAS9-D* have greater relative expression in roots under Fe sufficiency than Fe deficiency. These genes are closely related to *OsNAS3* and they might operate under Fe excess in the same manner as *OsNAS3*. Expression of Clade II *NAS* genes decreased under Fe deficiency and was induced in roots and shoots under normal Fe condition. Thus, Clade II NAS proteins may not contribute to MA biosynthesis under Fe deficiency, but may be involved in NA biosynthesis to support Fe loading of vascular tissues and maintenance of cellular Fe homeostasis (Bonneau et al., 2016).

### The *OsNAS3* Knockout Plants Are Sensitive to Fe Excess

The *OsNAS3* knockout plants resulted in impaired shoot and root growth than NT under both control and Fe excess conditions (Figure 7). The NT plants under Fe-excess set panicles likewise under control Fe condition but the knockout plants under Fe-excess did not set panicles (Figure 7A,B). Dry weights of the knockout plants reduced in both shoots and roots under excess Fe (Figure 7G,H). In this study, the leaf bronzing caused by Fe excess damage in knockout plants was more pronounced than the growth defect. Our results consistently indicated that the knockout line was more susceptible to Fe excess than NT plants in terms of leaf bronzing levels and Fe accumulation in the newest leaves and old leaves (third newest leaves) compared to NT plants (Figure 8, 9, 10). Disruption of *OsNAS3* clearly enhanced Fe accumulation in leaves in response to excess Fe, but only slightly higher Fe accumulation occurred in the roots than in NT roots (Figure 9A,E). These results suggest that enhanced Fe toxicity in *OsNAS3* knockout lines might be due to enhanced Fe translocation from roots to shoots. In fact, NA chelates excess Fe and supports its efficient translocation and sequestration, and hence the plant can reduce Fe translocation to shoots. However, high Fe accumulation was observed in Fe-excess shoots, showing that this knockout rice was unable to translocate excess Fe to the suitable tissues in roots. Iron uptake-related transporters such as *OsIRT1, OsIRT2, OsNRAMP1, OsYSL2*, and *OsYSL15* were highly suppressed under Fe excess (Aung et al., 2018). Thus, this high level of Fe accumulation in shoots may be due to Fe uptake by other chelators or other mineral transporters, for example, Zn transporters, which can take up Fe as well as Zn. These results indicate that *OsNAS3* plays an important role in mitigating Fe excess in rice.

In this study, the treatment applied was severe excess ferrous Fe stress (× 70 Fe; 2520 μM Fe^2+^) compared to the control in the hydroponic culture at low pH (pH 4.0). We also showed that the transgenic line with higher NA production showed tolerance to this severe level of Fe excess stress (Supplementary Figure S5). This line accumulated 15 times more NA and 3 times more DMA in its shoots (Masuda et al., 2009). These results suggest that increased NA and DMA biosynthesis might mitigate the damage from excess Fe in the plant.

### Role of NA in Fe Excess and Zn Deficiency

Iron excess leads to Zn deficiency. The Zn concentration was decreased under Fe excess in roots of both NT plants and the *OsNAS3* knockout line (Figure 9F). The concentrations of Zn decrease proportionally with the increase in Fe excess levels in roots (Aung et al., 2018). Preventing Fe uptake in response to Fe excess may reduce Zn uptake capacity. At the same time, expression of some putative Zn transporter genes such as *OsZIP4, OsZIP5, OsZIP7*, and *OsZIP9* is strongly induced in Fe excess roots, suggesting that these putative Zn transporters may participate in enhanced Zn transport under Fe excess (Aung et al., 2018). Nicotianamine plays a vital role in intercellular and long-distance transport of Zn to maintain Zn homeostasis in plants (Clemens et al., 2013). The Zn concentration tended to decrease in new leaves and increased in old leaves in knockouts compared to NT plants under Fe excess conditions (Figure 9B). The knockout plant was also impaired in Zn translocation and distribution, suggesting that NA produced by *OsNAS3* may be important to maintaining Zn levels in the newest leaves with excess Fe. Interestingly, expression of *OsNAS3* increased in Zn-deficient roots and shoots, but not that of *OsNAS1* or *OsNAS2* (Suzuki et al., 2008). Under Zn deficiency, *OsNAS3* expression is strongly induced in almost all tissues, by about 3 to 8 times (unpublished data). By contrast, under excess Zn, *OsNAS3* was highly repressed in both roots and shoots, while *OsNAS1* and *OsNAS2* were highly induced (Ishimaru et al., 2008). In this study, in addition to NA, DMA was present in roots and shoots under Fe excess (Figure 6B). DMA increases Zn translocation in Zn-deficient rice (Suzuki et al., 2008). It indicates another important role of NA synthesized by *OsNAS3* under excess Fe conditions, alleviating Zn deficiency in rice.

## Conclusion

In this study, we provide evidence that *OsNAS3* is functional and physiologically crucial under excess Fe, acting as an iron-excess induced gene. Our results suggest that NA and DMA synthesized by *OsNAS3* under excess Fe conditions contribute to Fe detoxification in rice. Nicotianamine plays multiple important roles in Fe nutrition in plants, which can be applied to Fe detoxification as well as Fe deficiency tolerance and Fe biofortification. This finding will contribute to developing Fe toxicity tolerant rice for growth in acidic paddy fields, which has the potential for improving rice yield to feed the increasing global population.

## Author Contributions

MSA, HM, and NKN designed, led, and coordinated the overall study. MSA performed most of the experiments, analyzed the results, and wrote the manuscript with assistance from HM. TN analyzed the NA and DMA concentrations. GA and J-SJ provided *OsNAS3* knockout seeds. HM, TK, and NKN discussed the results and improved the manuscript.

## Conflict of Interest Statement

The authors declare that the research was conducted in the absence of any commercial or financial relationships that could be construed as a potential conflict of interest.
