# Supplementary material for: Nicotianamine Synthesis by OsNAS3 Is Important for Mitigating Iron Excess Stress in Rice
Source: Front Plant Sci. 2019 Jun 4;10:660. doi: 10.3389/fpls.2019.00660 (PMC6558524; doi:10.3389/fpls.2019.00660)
Supplement: Supplementary file 1 [file Data_Sheet_1.pdf]

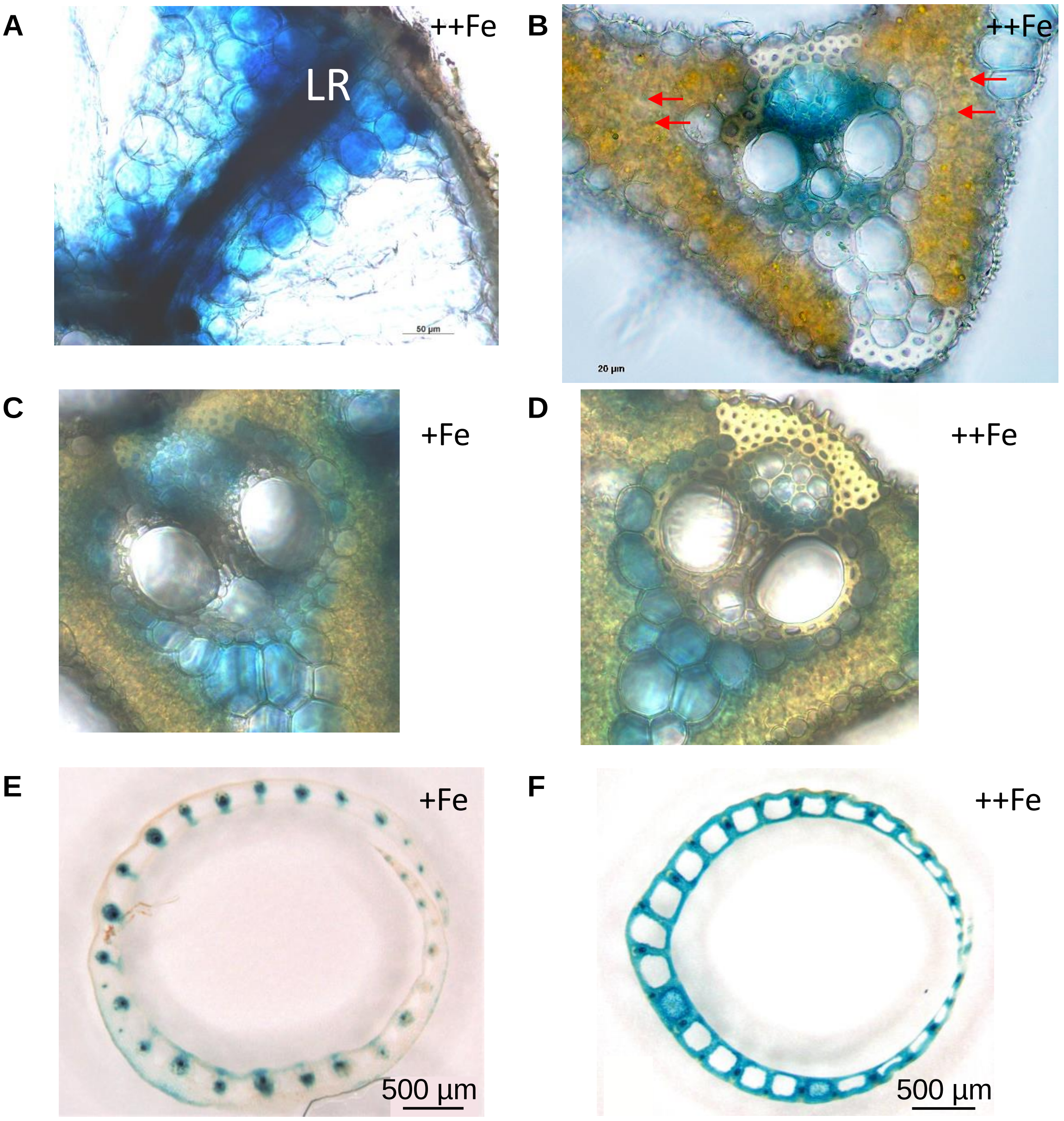

SUPPLEMENTARY FIGURE 1 | Cellular localization of *35S* promoter-*OsNAS3* GUS expression in transgenic rice plants. **(A)** Lateral roots of the basal portion of mature roots under Fe excess. **(B)** Fe-excess old leaves. Red arrows, chloroplasts inside mesophyll cells. **(C)** Control newest leaf. **(D)** Fe-excess newest leaf. **(E)** Outer layer of DC in control plant. **(F)** Outer layer of Dc in Fe-excess plant. Scale bars: 20 µm for **B,C,D**; 50 µm for **A**; 500 µm for **E,F**.

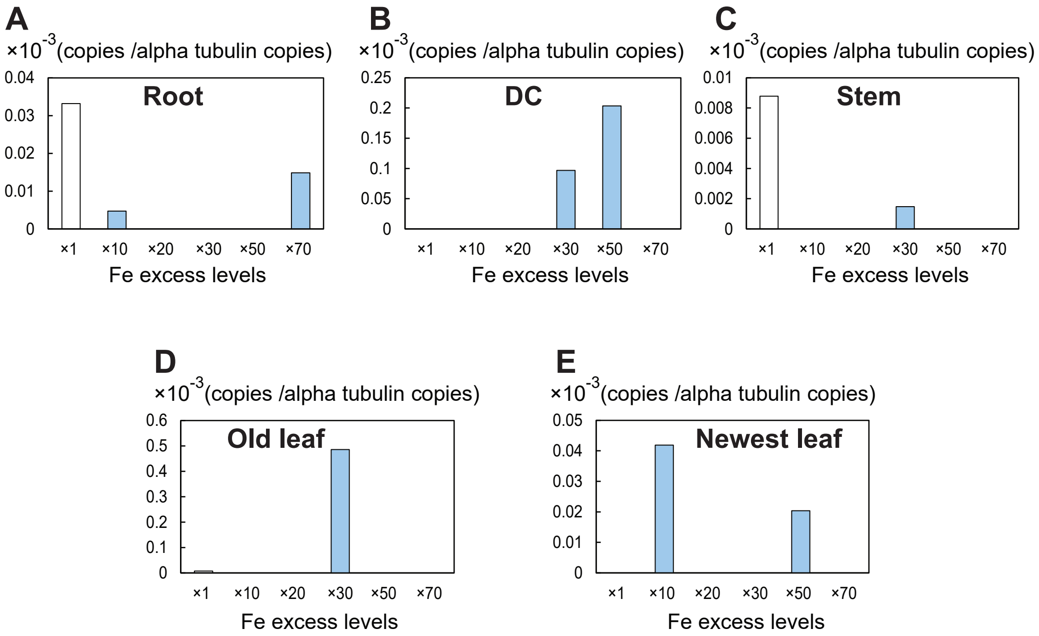

SUPPLEMENTARY FIGURE 2 | Expression levels of the *OsNAAT1* gene in rice tissues under control and various Fe excess conditions. **(A)** Roots. **(B)** DCs. **(C)** Stems. **(D)** Old leaves. **(E)** Newest leaves. DC: discrimination center. This figure shows confirmation of the microarray results listed in Table 1 of Aung et al. (2018) from qPCR analyses. Transcript levels were normalized to the expression levels of alpha-*tubulin* determined using the primers of alpha-*Tubulin*.

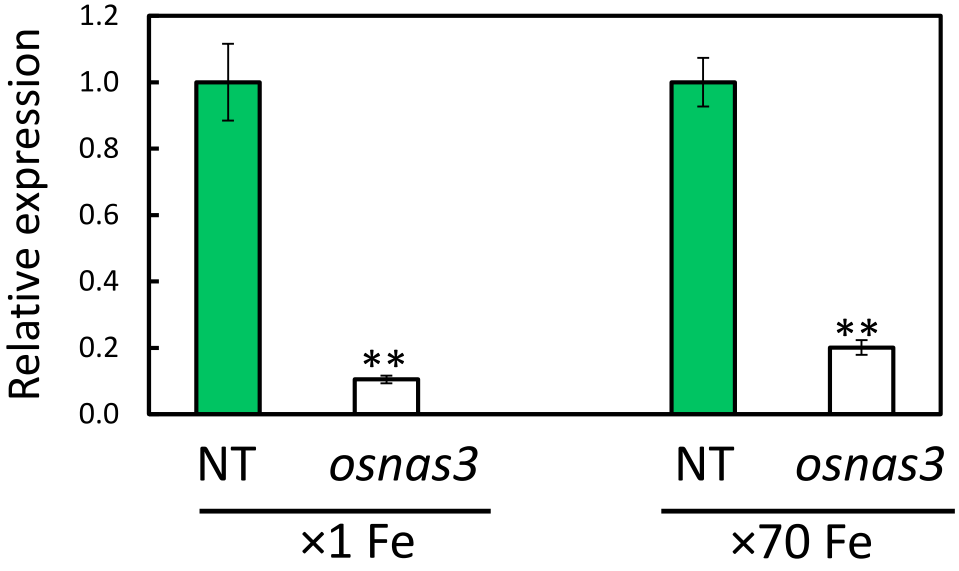

SUPPLEMENTARY FIGURE 3 | Expression of *OsNAS3* knockout plants and NT determined through qPCR analyses. Error bars represent ± 1 SE of technical replicates, n = 3. Data were normalized to the observed expression levels of *alpha-Tubulin* and presented as relative gene expression in each tissue (×1 Fe = 1). Asterisks above the bars indicate significant differences (**P* < 0.05; ***P* < 0.01) compared to control (×1 Fe).

A

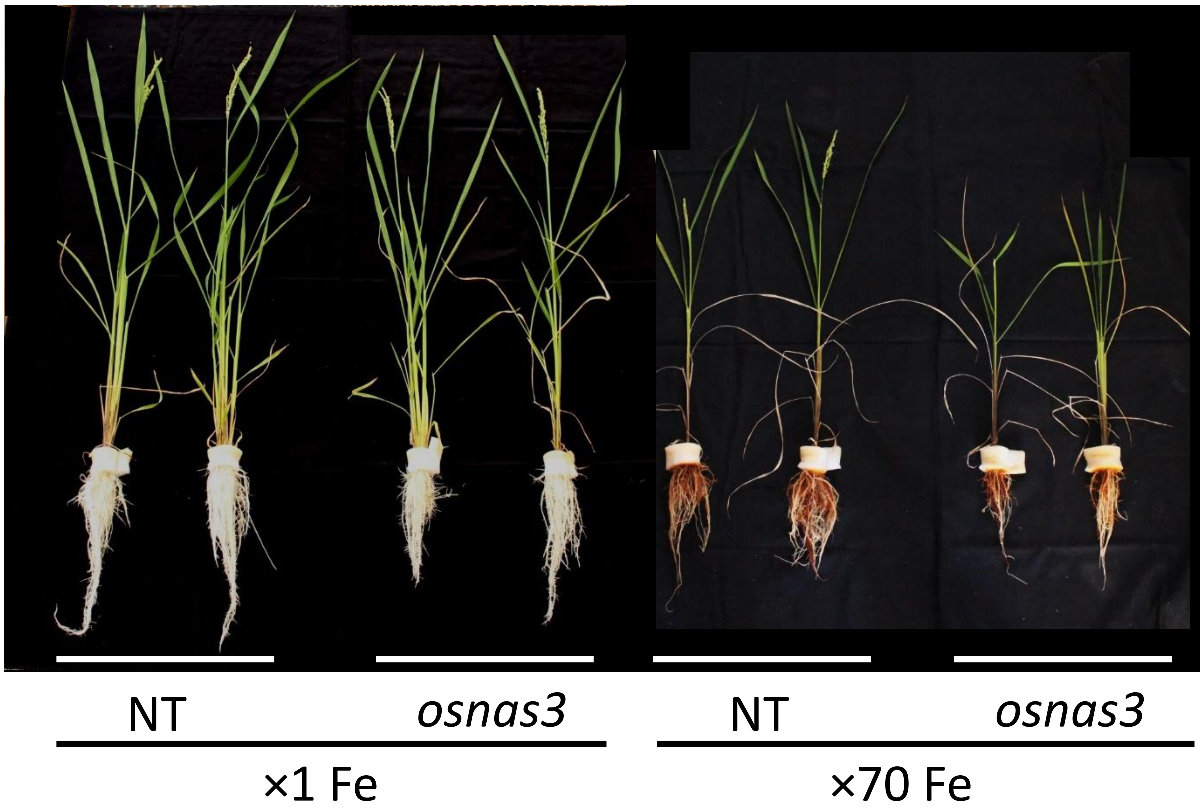

B

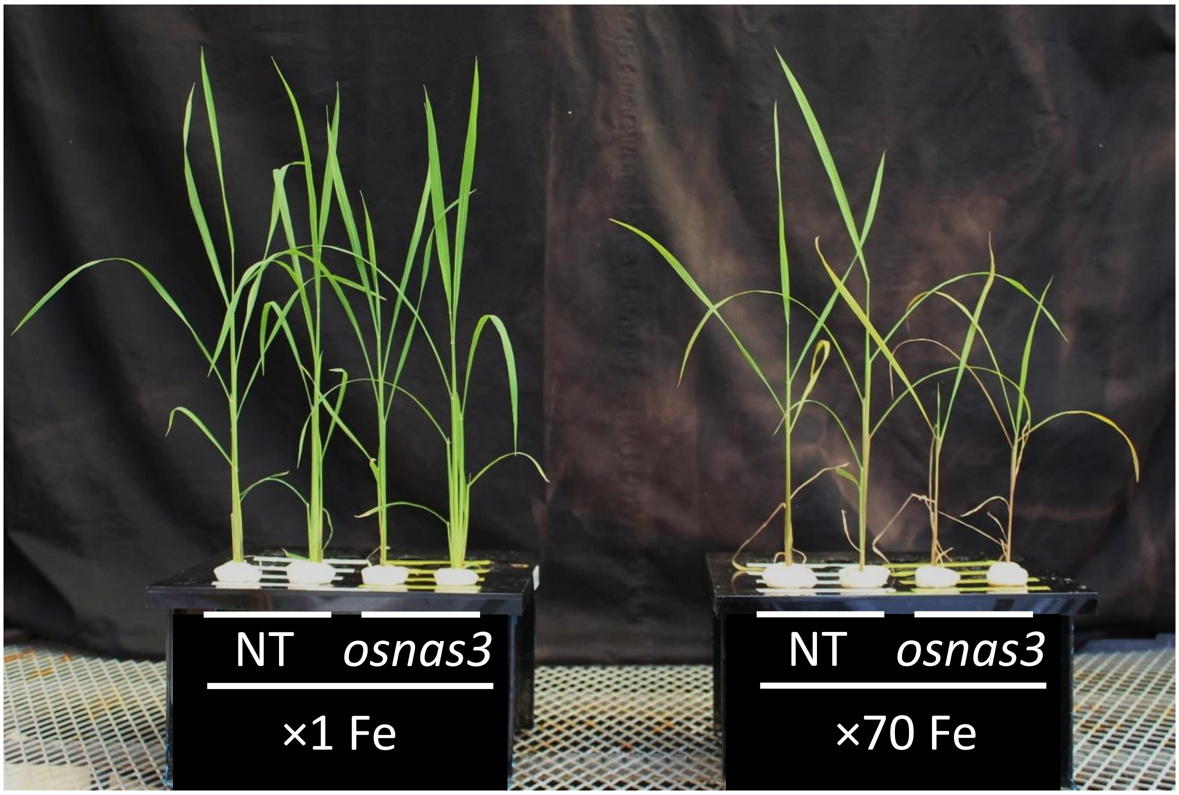

SUPPLEMENTARY FIGURE 4 | Plant appearance of NT and *OsNAS3* knockout plants under normal and excess Fe conditions. **(A)** First experiment after 39 days of Fe excess exposure. **(B)** Second experiment after 23 days of Fe excess exposure.

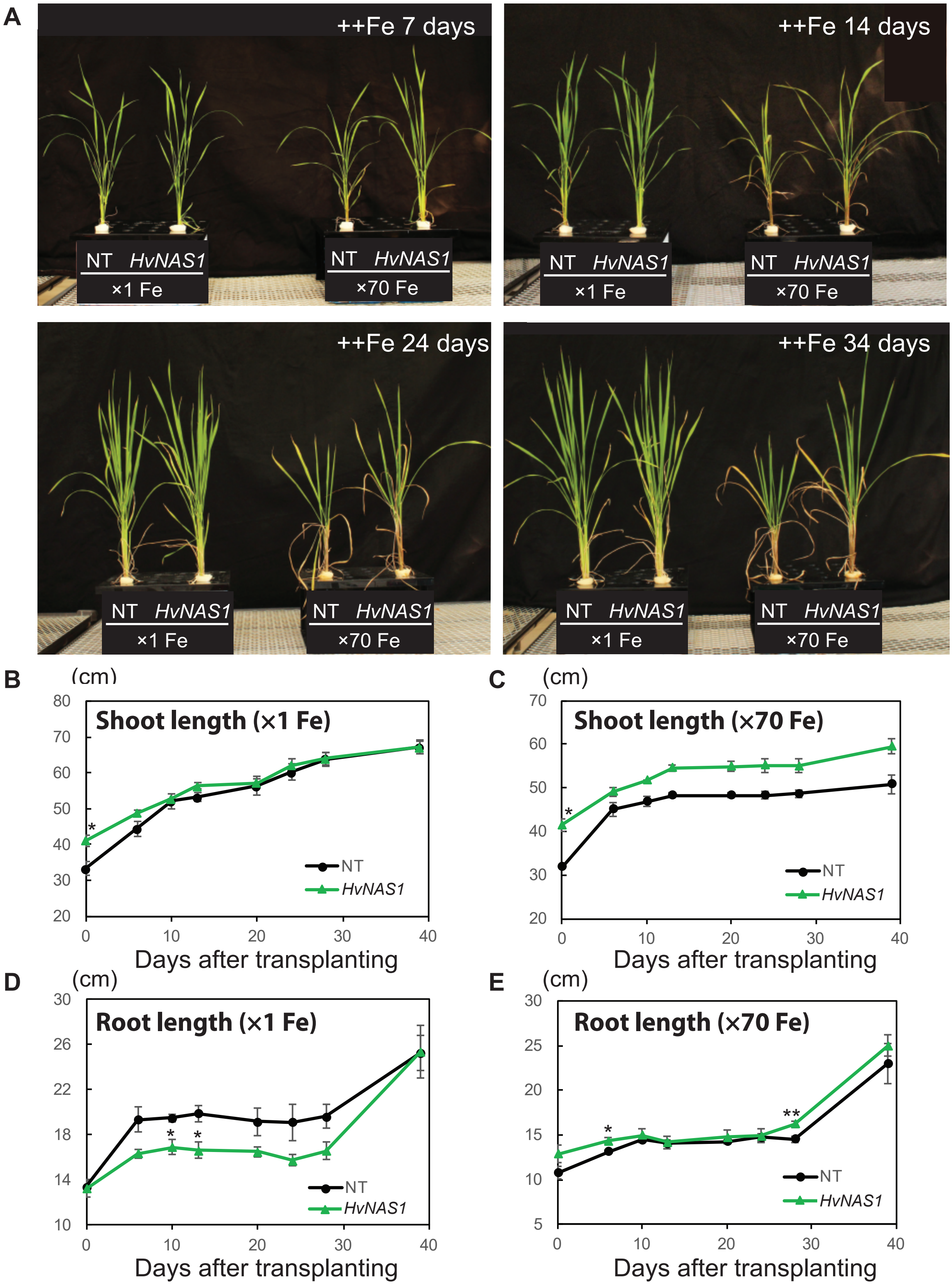

SUPPLEMENTARY FIGURE 5 | Plant appearance and growth of elevated NA production rice and non-transformant (NT). **(A)** Appearance of NAS-overexpressing and NT plants under control or Fe excess after 7, 14, 24, and 34 days. **(B,C)** Shoot lengths and **(D,E)** Root lengths under control and excess Fe. Error bars represent the standard error (SE) of biological replicates, n = 4 for NT and n = 3 for overexpressing plants. Plants were grown hydroponically under control (×1 Fe) and excess ferrous Fe (×70 Fe) conditions at pH 4.0. Asterisks indicate significant differences compared to the NT at each time point (*$P < 0.05$, **$P < 0.01$).
